# Supplementary material for: Accuracy Assessment of the GlucoMen® Day CGM System in Individuals with Type 1 Diabetes: A Pilot Study
Source: Biosensors (Basel). 2022 Feb 9;12(2):106. doi: 10.3390/bios12020106 (PMC8869704; doi:10.3390/bios12020106)
Supplement: Supplementary file 1 [file biosensors-12-00106-s001.zip › biosensors-1479473-supplementary.pdf]

biosensors

MDPI

Article

Accuracy Assessment of the GlucoMen® Day CGM System in Individuals with Type 1 Diabetes: A Pilot Study

Daniel A. Hochfellner, Amra Simic, Marlene T. Taucher, Lea S. Sailer, Julia Kopanz, Tina Pöttler and Julia K. Mader *

Division of Endocrinology and Diabetology, Department of Internal Medicine, Medical University of Graz, Graz, Austria; daniel.hochfellner@medunigraz.at (D.A.H.); amra.ajsic@medunigraz.at (A.S.); marlenetaucher@gmail.com (M.T.T.); lea.sailer02@gmail.com (L.S.S.); juliakopanz@gmx.at (J.K.); tina.poettler@medunigraz.at (T.P.)

* Correspondence: julia.mader@medunigraz.at; Tel.: +0043-316-385-12383

Supplementary Materials:

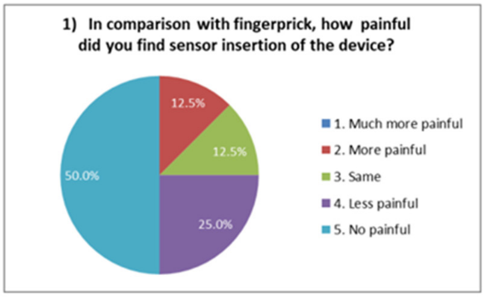

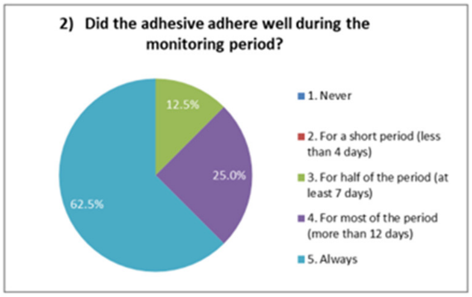

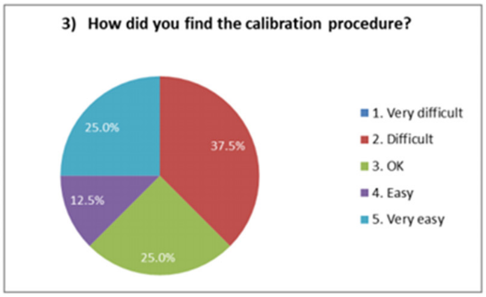

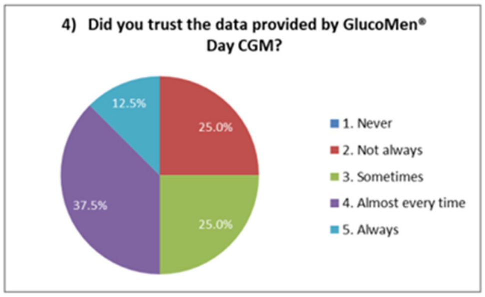

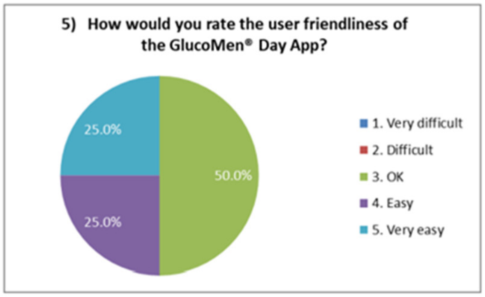

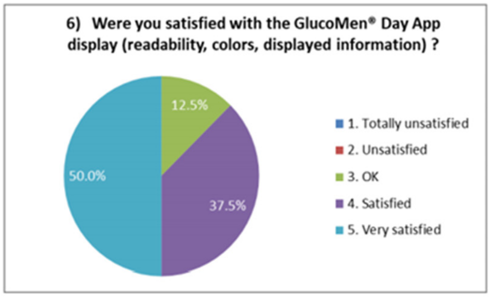

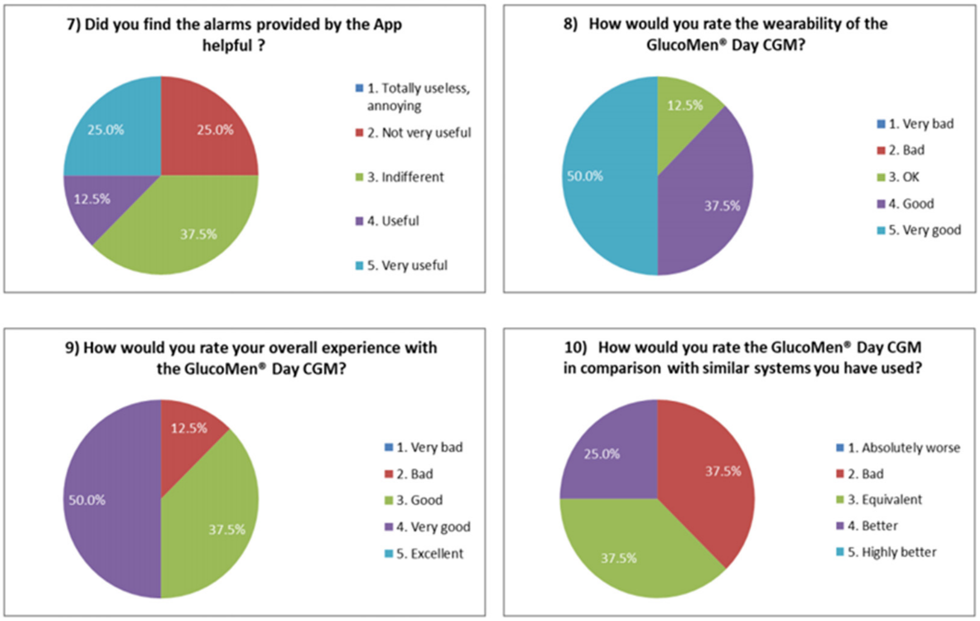

Figure S1. Usability Questionnaire Results (N = 8).
